# Peri-implant tissue response to three implants engaging retained roots: a histologic report in the pig mandible

**DOI:** 10.1186/s40729-026-00676-4

**Published:** 2026-04-06

**Authors:** Serge Szmukler-Moncler, Roni Kolerman, Nirit Tagger-Green, Florian Beuer, Stefan Milicescu

**Affiliations:** 1 Department of Prosthodontics, Geriatric Dentistry and Craniomandibular Disorders, Charité University of Medicine, Charité Center 03, Assmannshauser Str. 4-6, 14197 Berlin, Germany; 2 Berlin Implantology Research Group, Eichhornstrasse 2, 10785 Berlin, Germany; 3 Department of Periodontology, Maurice and Gabriela Goldschleger School of Dental Medicine, Tel Aviv University, Tel Aviv, Israel; 4 Department of Esthetic Dentistry, Faculty of Dentistry, Carol Davila University of Medicine, Bucarest, Romania

## Abstract

**Purpose:**

The biological response of dental tissues to implants placed in contact with retained roots is poorly documented. The aim of this report is to describe the histological findings of three implants that inadvertently engaged inflammation-free retained root fragments during placement and were retrieved after 10 and 12 weeks of healing in the pig mandible.

**Materials and methods:**

Three implants were retrieved from a preclinical pig model in which fractured root remnants were inadvertently left in situ following tooth extraction; implants were placed after a three-month healing period and were harvested after 10 and 12 weeks. Histological evaluation was carried out using optical microscopy, polarized light microscopy, and scanning electron microscopy coupled with energy-dispersive X-ray spectroscopy to characterize the tissue responses at the implant–root interface. Three configurations of implant–root contact were identified: (1) an implant fully embedded within a retained root composed of dentin and osteodentin; (2) an implant presenting apical contact with a retained root; and (3) an implant apex engaging fractured-root debris.

**Results:**

The implant fully embedded within the retained root exhibited uninterrupted contact with dentin and osteodentin, with no intervening soft-tissue layer. Areas of tight implant–dentin apposition were devoid of cellular activity; conversely, in regions where microgaps or interfacial space were present, osteodentin showed active modeling and remodeling along the implant surface. In the second and third samples, osseointegration was observed along the implant body in contact with bone despite the presence of apical contact with residual root tissues. Newly formed osteodentin was intimately adapted to the implant apex, whereas the periodontal ligament that persisted did not extend appreciably onto the implant surface.

**Conclusions:**

This preclinical report describes the histological response to three implants in contact with non-infected retained roots. In two specimens with partial root contact, bone was present at the interface and osseointegration was observed. In all three specimens, newly formed osteodentin was present at the implant interface, with evidence of early modeling and remodeling. Further controlled investigations are required to confirm these observations and to assess their reproducibility and broader biological relevance.

## Background

Dental implant therapy is a well-established and widely accepted modality for the replacement of missing teeth; it offers high survival rates and predictable long-term outcomes [1]. Standard protocols involve placement into healed alveolar bone or into fresh extraction sockets. In clinical practice, however, clinicians may encounter retained roots or root fragments left behind after incomplete extractions along the planned implant trajectory.

In edentulous patients, the prevalence of such retained dental structures is not negligible, particularly in the maxilla; reported frequencies are ranging from 15.4–37.3% [2–4]. In partially dentate patients, lower incidence rates have been documented, typically between 11.3% and 20% [5]. This scenario is becoming increasingly frequent with the growing adoption of techniques such as the submerged root concept [6–8] and decoronation [9, 10]; both approaches intentionally preserve root structures below the crest to maintain alveolar bone volume.

When a retained root is identified, the clinical team must determine whether to remove it or whether to leave it *in situ.* A careful risk–benefit assessment is essential; surgical retrieval may require invasive procedures associated with increased morbidity, whereas asymptomatic retained roots may not warrant intervention. In their review, Nayyar et al. [5] emphasized that most retained roots remain asymptomatic, and their removal often entails extensive surgery that may not be clinically justified.

What occurs when an implant inadvertently encroaches upon a retained root left behind after a failed extraction remains poorly documented in the literature. Szmukler-Moncler et al. [11] reported six successfully rehabilitated patients with seven implants, followed for periods ranging from 20 months to 9 years; in contrast, a similar series by Langer et al. [12], involving seven implants in six patients, described multiple late failures occurring up to four years after loading, with infections arising long after placement and restoration. Three additional cases of implant failure under comparable circumstances have also been published [13–15]. The authors of these reports [12, 14] underscored the need for rigorous long-term evaluation before considering intentional retention of root fragments as a safe and predictable strategy, particularly in situations with a history of infection or pre-existing bone loss.

Histological insights into the consequences of implant–root contact do exist, but they remain scarce; nonetheless, they provide valuable information regarding tissue reactions at these unconventional interfaces. The available evidence can be divided into two groups: (1) human case reports in which implants were retrieved following clinical failure [12–15], and (2) animal studies in which implant–root contact was either intentionally created [16, 17] or identified retrospectively after healing [18, 19].

Preclinical protocols evaluating the histological characteristics of the bone–implant interface typically involve tooth extraction, a defined healing period, and subsequent implant placement. However, implants may occasionally be inadvertently positioned in contact with residual root fragments [18, 19]; although unintentional, such events offer a unique opportunity to examine, histologically, the implant–root interface beyond conventional osseointegration. With this perspective, we reviewed archived histologic sections from previous preclinical studies in which these unexpected findings occurred.

Notably, implants infringing upon a healed retained root should be clearly distinguished from implants encroaching upon adjacent vital teeth or from those contacting buccal root segments in the context of the socket-shield technique (SST) [20–22]; the underlying tissues and biological conditions differ substantially, and the resulting tissue responses may therefore not be comparable [19].

The aim of the present histological report is to describe the tissue response associated with implant placement in contact with retained roots. Specifically, it examines three implants from archived preclinical studies that had been placed into sites of fractured extractions; all implants were inserted in the mandible of the domestic pig and were retrieved after 10 and 12 weeks of healing.

## Materials and methods

### Origin of the samples

The histological material analyzed in this report originates from two independent preclinical studies conducted in the mandible of the Landrace pig; three adult female pigs weighing 180–200 kg were included [23], and the experimental protocols were approved by INRA-Dijon (Institut National de Recherche pour l’Agriculture, Dijon, France).

Objective of the first study was to evaluate the osseointegration of TG Osseotite implants (3i, Palm Beach Gardens, FL, USA) measuring Ø 4.0 × 10 mm after 2 and 10 weeks of healing. These implants are manufactured from titanium grade 5 (Ti-6Al-4 V) and feature a moderately etched surface; the resulting microtopography exhibits micropits that promote bone ingrowth, with an average roughness (Ra) of < 1 μm [24].

The canines and premolars were extracted in both sides; this provided a 90–100 mm space per hemi-mandible for implant insertion. After 3 months of post-extraction healing, the animals were anesthetized under standard veterinary general anesthesia protocols. Full-thickness mucoperiosteal flaps were elevated to expose the edentulous ridge. Osteotomies were prepared using sequential drilling under copious sterile saline irrigation according to the manufacturer’s protocol. The implants were inserted and all achieved primary stability. Cover screws were placed, and the implants were left to heal in a submerged manner instead of transgingival because dental hygiene cannot be performed in this animal model without anesthesia [23]. The flaps were repositioned and sutured with interrupted resorbable sutures. Postoperative management followed institutional veterinary guidelines.

After 2 and 10 weeks of healing, the animals were anesthetized; blocks containing the implants were obtained through segmental osteotomy. They were fixed in 10% buffered formalin and each implant site was then processed for non-decalcified histological sectioning. The samples were then dehydrated in an ascending series of alcohol rinses and embedded in glycolmethacrylate resin (Technovit 7200 VLC, Kulzer, Germany). After polymerization, vestibulo-lingual sections of about 150 μm in thickness were prepared; they were then ground to 30–50 μm thick sections. Central slices of each implant were stained with Paragon for histological evaluation.

The roots of the domestic pig are thin and fragile; several fractured during extraction and remained in situ. Histological examination of all the 10-week specimens revealed that one implant out of 12 had been inadvertently placed through a retained root, while another section showed a retained root located at some distance from the implant.

The second study involved TPS-coated ITI implants (Ø 4.1 × 10 mm) placed in the mandible of the same domestic pig model [25]. These implants are made of titanium grade 4 and feature a rough titanium–plasma-sprayed (TPS) surface with an Ra > 2 μm [24]. After a three-month healing period, the samples were retrieved and processed for histology using the same procedures described above. Histological examination of all the samples revealed two histological sections out of 12 in contact with retained root fragments.

#### Light microscopy (LM)

The histological sections were examined several years apart using a binocular light microscope (Olympus BX50, Olympus, Hamburg, Germany) equipped with a color CCD camera (Color View III, Olympus, Hamburg, Germany). Observations were performed under conventional bright-field light (LM) as well as polarized light (PLM), at magnifications ranging from ×2 to ×20.

#### Scanning electron microscope (SEM) and energy-dispersive X-ray spectroscopy (EDS)

The sections were examined several years apart using two different SEM–EDS systems; the aim was descriptive and qualitative rather than quantitative. Initial observations were performed using a Zeiss EVO 1450VP variable-pressure SEM (Carl Zeiss, Oberkochen, Germany) operated at 15 kV with a working distance of 17 mm and equipped with a Si(Li) EDS detector (Thermo Electron NORAN, Thermo Fisher Scientific, Waltham, MA, USA).

Subsequent analyses were carried out using an FEI Quanta 200 F (Thermo Fisher Scientific, Waltham, MA, USA), operated at 20 kV with a working distance of 10 mm, capable of environmental (ESEM) operation and coupled with an Oxford Instruments X-Paxn EDS system (Abingdon, UK).

Elemental mapping of calcium (Ca) and phosphorus (P) was performed with a 15-minute acquisition time over defined regions of interest (100 μm × 75 μm for the first system and 130 μm × 115 μm for the second). Regions of interest were selected based on previously identified histological implant–tissue interface areas.

All acquisition parameters were kept constant within each instrument configuration. Analyses were qualitative in nature and focused on elemental presence and spatial distribution; no quantitative or semi-quantitative elemental ratios were calculated.

## Results

### Implant #1 encased within a retained root, retrieved after 10 weeks

#### LM observation

**Fig. 1:**
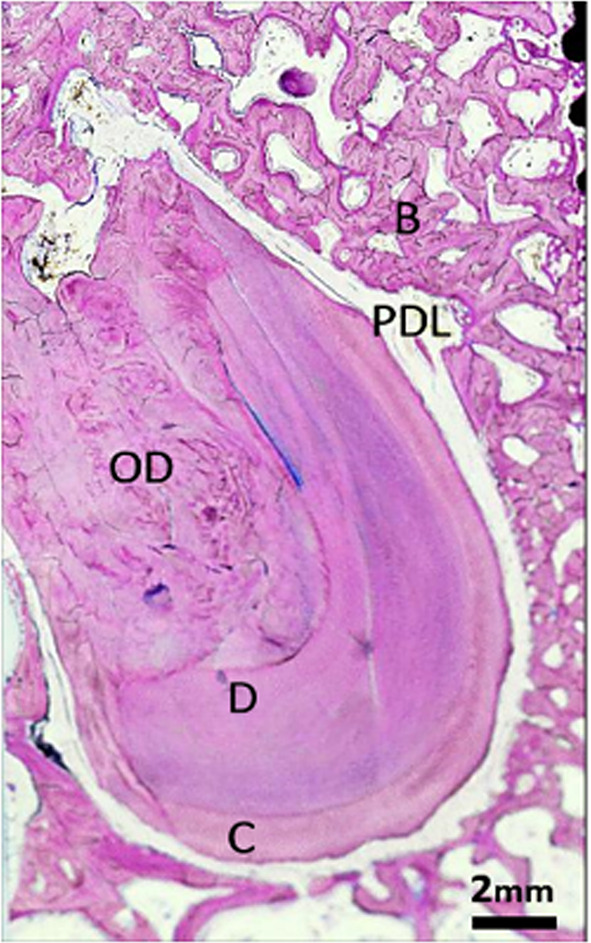
Histological image of a retained root. The section shows a shell composed of dentin and cementum, surrounded by the periodontal ligament (PDL), and filled with reactive tertiary reparative osteodentin. All sections in this report were stained with Paragon. *B* bone, *D* dentin, *C* cement, *OD* osteodentin, *PDL* periodontal ligament. (LM, objective x2, scale bar 2 mm)

The implant was placed within the remains of a fractured root that had undergone a reparative process during the 3-month healing period between partial extraction and implant placement. Figure 1 shows a retained root fragment from an adjacent histological section; the fragment consists of a shell of dentin and cementum surrounded by the intact periodontal ligament (PDL). This dentin–cementum shell was filled with reparative dentin, produced in response to the severe trauma and pulp exposure caused by the root fracture [26, 27].

Under light microscopy, dentine was identified by the presence of regular dentinal tubules radiating from the pulp toward the periphery. Under polarized light, it exhibited a strong, highly organized birefringent pattern consistent with the parallel orientation of its collagen matrix [26, 27].

Osteodentin was distinguished by its disorganized matrix and the presence of entrapped cells within lacunae, resembling woven bone [26, 27]. In polarized light, it demonstrated an irregular, woven birefringence lacking the directional tubular organization characteristic of dentine. Osteodentin formed during the healing period before implant placement was labelled OD1 and osteodentin formed afterwards was labelled OD2.

Cementum was identified on the external root surface by the absence of dentinal tubules, the presence of incremental lines. Under polarized light, it exhibited a moderate birefringent pattern with collagen fibers oriented predominantly parallel to the root surface.

Neocementum was defined as newly deposited mineralized tissue lacking dentinal tubules and located either on exposed dentine or directly on the implant surface in continuity with cementum. In polarized light, it displayed a heterogeneous and often less organized birefringent pattern.

**Fig. 2:**
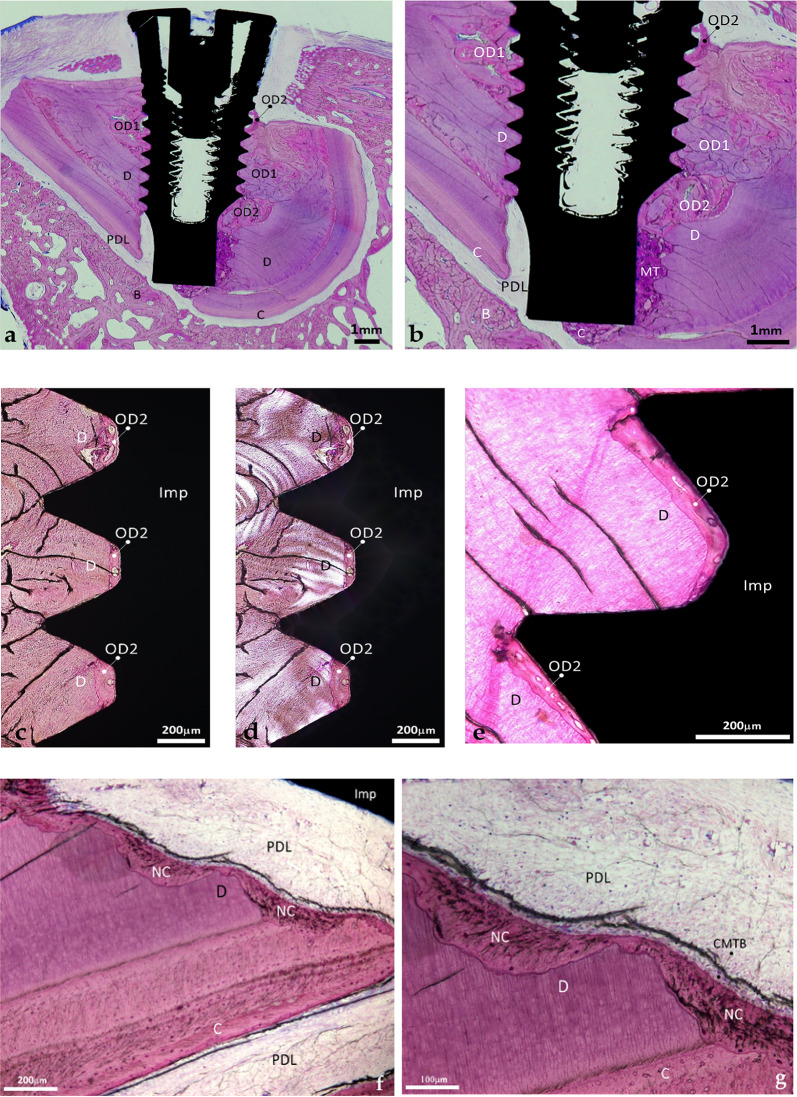
Histological section of an implant fully encased in a retained root after a 10-week healing period. **a** Overview of the implant and the retained root. Bone is absent at the most coronal level due to inflammation associated with suture dehiscence. Note the pristine dentine and cementum, the PDL and the newly formed reactive tissues. Cracks observed in the histological section are attributed to the prolonged interval between specimen preparation and microscopic examination (LM, objective x4, scale bar 1 mm). **b** Close-up of figure **a**. It provides clearer identification of the various tissues in contact with the implant. *D* dentine, *C* cement, *PDL* Periodontal ligament, *B* bone, *OD1* osteodentin formed after tooth extraction, *OD2* osteodentin formed after implant placement, *MT* mineralized tissue with limited presence of collagen (LM, objective x4, scale bar 1 mm). **c** Pristine dentine and neoformed osteodentin within the valleys of the implant threads. Osteodentin was formed in the space defined by the valleys and remodeled the adjacent dentine. (LM, objective x10, scale bar 200 μm). **d** Observation of the previous section under polarized light. The birefringence patterns differ between the original dentin and the newly formed tissue occupying the thread valleys (PLM, objective x10, scale bar 200 μm). **e** Neoformation of a mineralized tissue in the spaces along the implant threads. *D* dentine, *OD2* osteodentin formed after implant placement, *Imp* implant (LM, objective x20, scale bar 200 μm). **f** Resorption of the dentin in contact with the conjunctive tissue of the PDL accompanied by the apposition of newly formed cementum (LM, objective x20, scale bar 200 μm). **g** Close-up of figure (f) showing the active secretory activity of cementoblasts in direct contact with the PDL. *D* dentine, *C* cementum, *NC* neocement, *PDL* periodontal ligament, *CMTB* cementoblasts (LM, objective x20, scale bar 100 μm)

Figure 2a, b show the implant completely encased within a retained root. The absence of bone around the coronal microthreads reflects postoperative inflammation secondary to suture dehiscence; histological signs of bone repair were evident, and the inflammatory process did not extend apically toward the retained root. The numerous cracks observed in the section are artifacts related to the prolonged interval between specimen preparation and microscopic examination.

The implant threads came in contact with the pristine dentin, with the osteodentin formed during the post-extraction healing period, and with the newly deposited osteodentin that developed after implant insertion, as indicated by its deeper staining. Along the threads, the dentin in close contact with the implant surface did not show signs of tissue remodeling (Fig. 2c–e). In contrast, the voids left between the implant surface and the dentin, in the valleys between the threads, were filled with a tissue that displayed marrow-like spaces and initiated remodeling of the adjacent dentin (Fig. 2c–e).

At the apex, the hollow region caused by drilling and tapping, initially in contact with the PDL, was infiltrated by a PDL-derived connective tissue (Fig. 2b) which extended coronally until reaching the dentin. The dentin exposed to the conjunctive tissue showed signs of resorption; it was subsequently covered by a layer of neocement (Fig. 2e, f) with cementoblasts displaying a marked secretory activity (Fig. 2f).

**Fig. 3:**
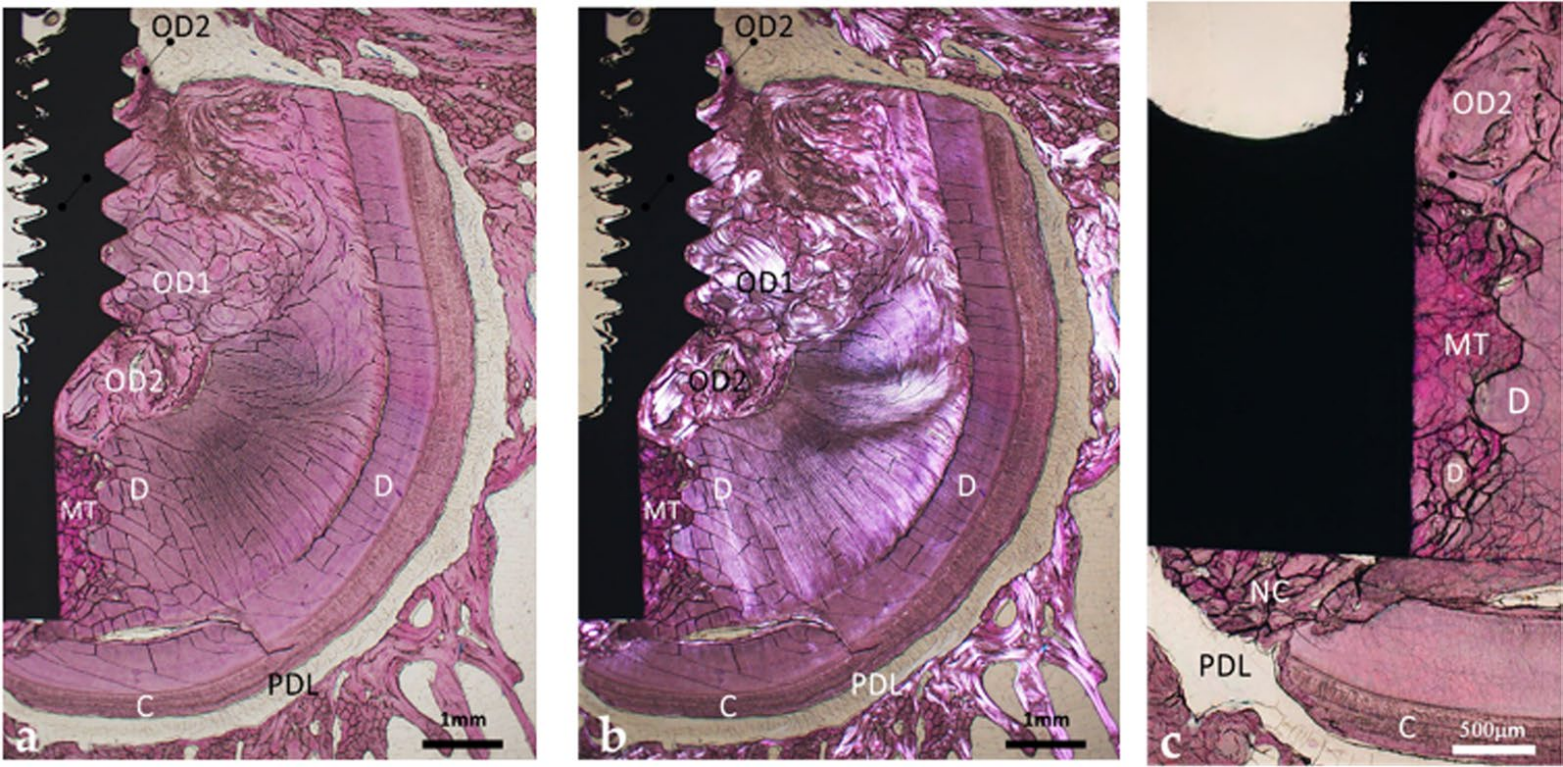
Histological view of the lingual side of the implant fully encased in the retained root. **a** Overview of the tissues in contact with the implant and at a distance. Note the distinct staining patterns of the pristine and newly formed root tissues (LM, objective x2, scale bar 1 mm). **b** Polarized-light view of figure (**a**). The birefringence varies according to tissue type; the disordered organization of the collagen matrix in the tissues formed after root injury is evident (PLM, objective x2, scale bar 1 mm). **c** Detailed view of the newly formed tissues in contact with the beveled apex of the implant. The staining highlights their respective structures: the concentric organization of the osteodentin at the coronal aspect of the bevel; the poorly organized tissue adjacent to the vertical portion of the apex; the partially organized tissue at the base of the apex; and the surrounding pristine dentin and cementum (LM, objective x4, scale bar 500 μm). *D* dentin, *C* cementum, *PDL* Periodontal ligament, *OD1* osteodentin formed after extraction, *OD2* osteodentin formed after implant placement, *MT* mineralized tissue with limited presence of collagen

On the opposite side of the implant (Figs. 2a and b and 3a–d), the coronal portion of the void space defined by the bevel was filled with a mixture of newly formed osteodentin and fragments of pristine dentin. This newly formed osteodentin displayed active modeling and remodeling both along the implant surface and at a distance from it (Figs. 2b and 3a–d).

More apically, the hollow space between the apex and the dentin, shaped by the taping threads, was filled with a mixture of dentin debris embedded in a mineralized matrix (MT, mineralized tissue) distinct from osteodentin; this mineralized matrix displayed a lower degree of collagen organization (Figs. 2b and 3a–d). The tissue adjacent to the apex shared morphological features with the newly deposited tissue observed at the base of the apex (Figs. 2b and 3a–d); the latter was in continuity with the cementum underlying the dentin (Fig. 3c). Under polarized light, the structural differences between original dentin, cementum, and neoformed tissues were evidenced (Figs. 2d and 3b–d). No soft tissue interposition was observed along the implant surface; instead, the implant was entirely surrounded by a combination of pristine and neoformed mineralized tissues.

#### SEM-EDS observation

**Fig. 4:**
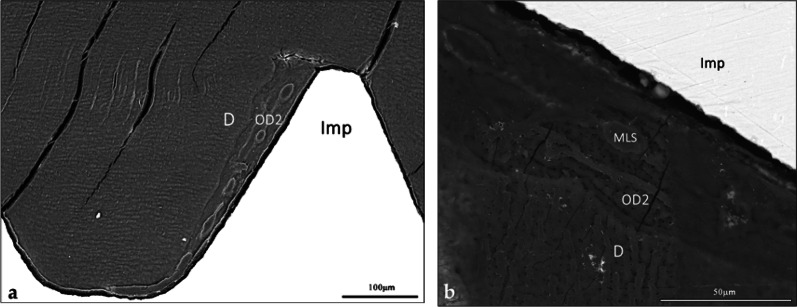
BSE-SEM micrographs of a thread presenting a microgap at one of the slopes. **a** Micrograph showing the native dentin and the neoformed mineralized tissue occupying the microgap and displaying signs of remodeling of the adjacent dentin (SEM, x200, scale bar 100 μm). **b** Higher-magnification of the microgap. Note the presence of tubules in the neoformed tissue oriented perpendicularly to those of the native dentin. The presence of tubules and marrow-like spaces characterizes this tissue as osteodentin (SEM, x750, scale bar 50 μm). *D* dentin, *OD2* osteodentin formed after implant placement, *MLS* marrow-like space, *Imp* implant

Scanning electron micrographs confirmed that the implant threads were entirely encased in mineralized tissue, either pristine dentin with characteristic tubules or newly formed tissue occupying the gaps between the implant and dentin (Fig. 4a, b). The junction between native dentin and the neoformed tissue was continuous (Fig. 4b). The neoformed tissue displayed a tubular structure perpendicular to the direction of the native dentine (Fig. 4b); it was consistent with organized tertiary dentine [27].

**Fig. 5:**
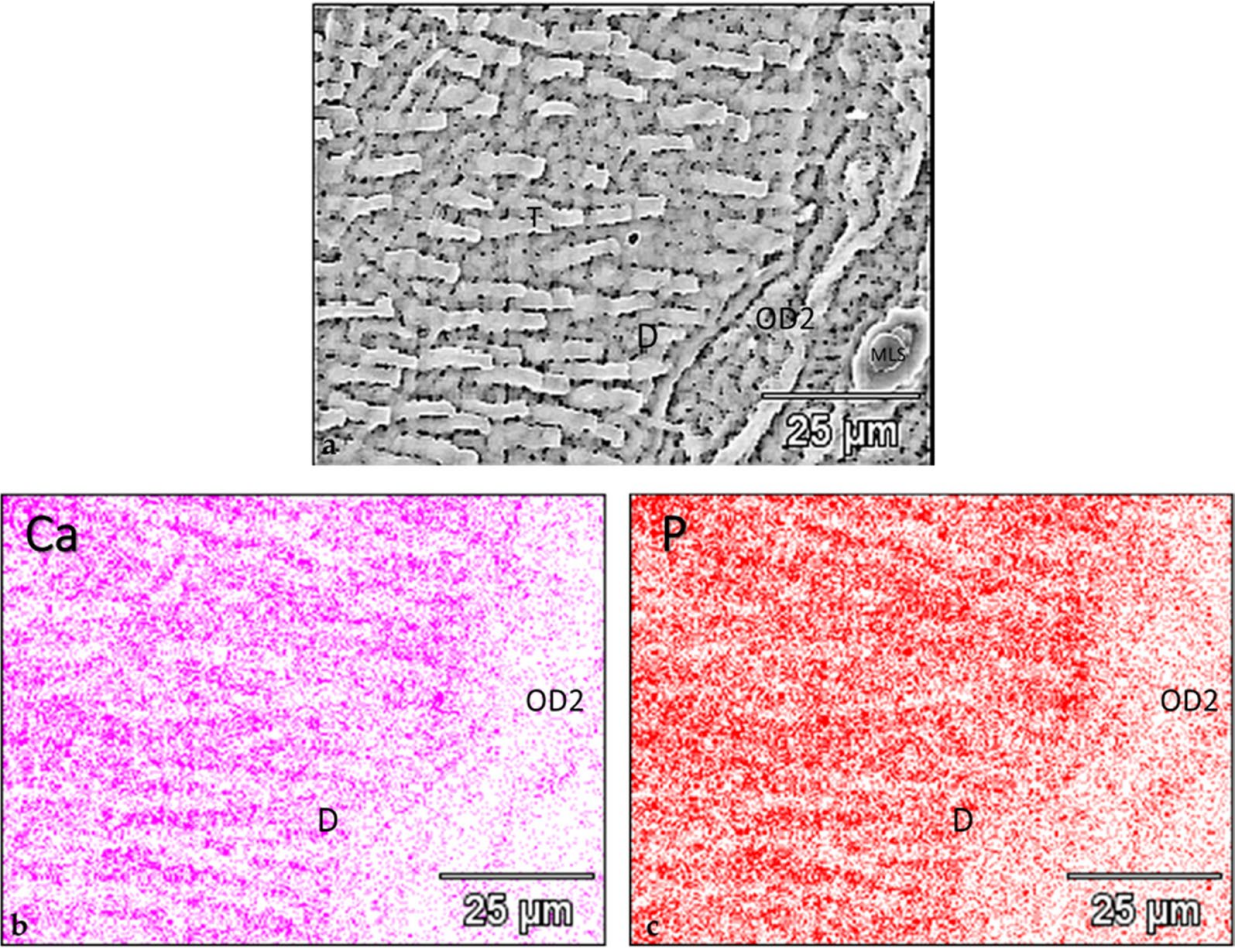
Elemental mapping of Ca and P at the dentin-osteodentin interface. **a** SEM micrograph in the back-scattered mode of the dentin and the neoformed tissue in the microgap. Note the distinct orientation of the tubules of the dentin and of the osteodentin, and the marrow-like space. Presence of tubules and marrow-like spaces characterizes this tissue as osteodentin (SEM, x1000, scale bar 25 μm). **b** Ca mapping of the dentin-osteodentin interface. The neoformed tissue shows a reduced signal intensity compared to the adjacent dentin. **c** P mapping of the same interface. The neoformed tissue shows a reduced signal intensity as well. *D* dentin, *OD2* osteodentin formed after implant placement

Elemental mapping at the interface between the native and neoformed tissues revealed a reduced Ca and P signal intensity in the newly grown tissue (Fig. 5a–c);

### Implant #2 in contact with a retained root, retrieved after 12 weeks

#### LM observation

**Fig. 6:**
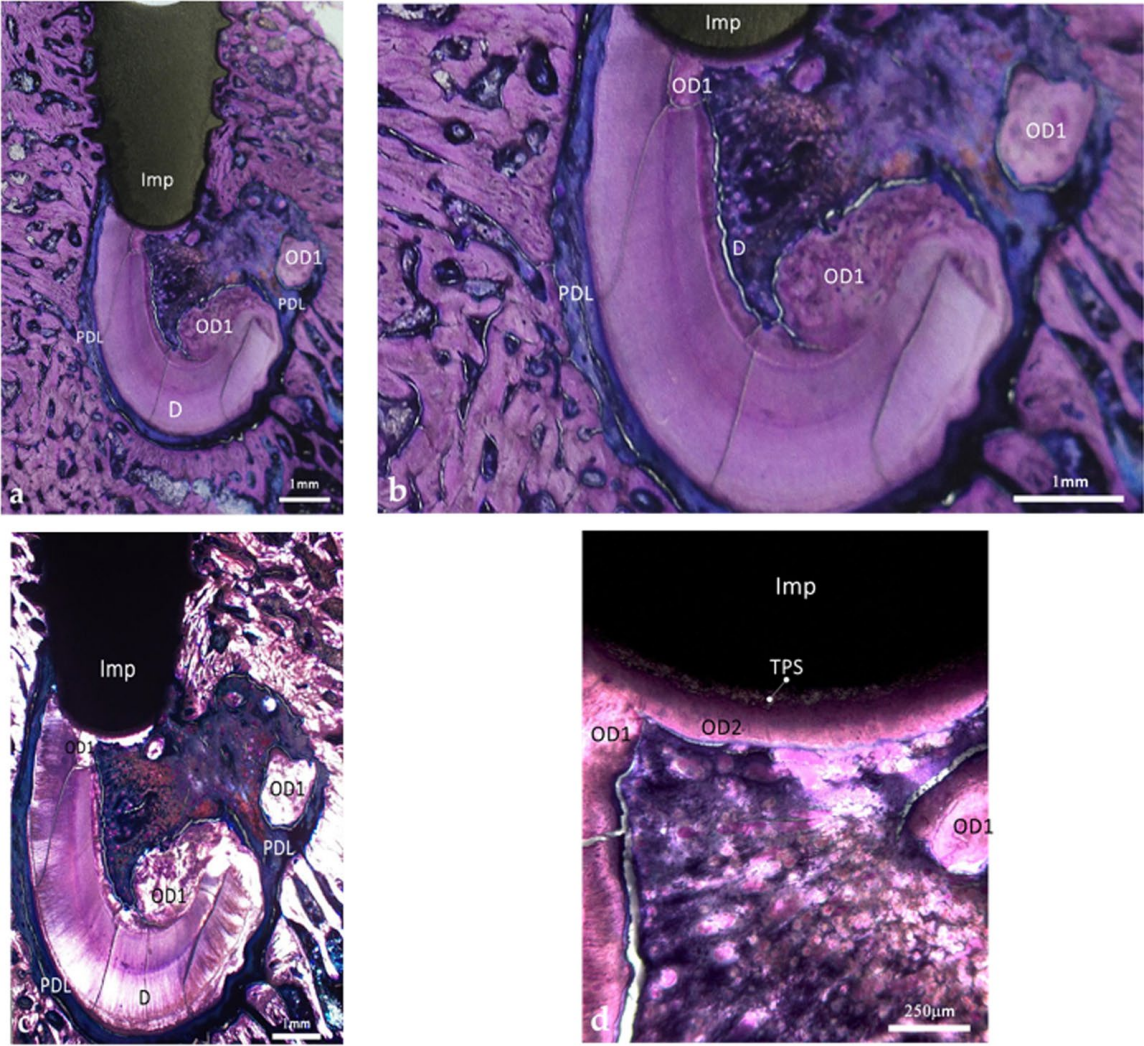
Histological micrograph of the second implant in contact with a retained root. **a** Only the apical portion of the implant contacts the retained root. Note the implant threads in contact with spongious bone, the shell of the root with the dentin, the reparative tissue, the PDL and the extended conjunctive tissue (LM, objective x2, scale bar 1 mm). **b** Close-up of the previous view showing the neoformed tissue in contact with the apex (LM, objective x4, scale bar 1 mm). **c** Observation under polarized light of figure a. The birefringence is different for every type of tissue. Note the distinct organization of the collagen matrix of the various mineralized tissues (PLM, objective x2, scale bar 1 mm). **d** Close up at the apex of the implant showing the close apposition of osteodentin at the surface of the apex (PLM, objective x10, scale bar 250 μm). *D* dentin, *OD1* osteodentin formed after extraction, *PDL* periodontal ligament, *TPS* rough titanium plasma-sprayed surface, *Imp* implant

This implant, retrieved after a 12-week healing period, came into limited contact with the retained root; unlike the first case, root contact was restricted to the implant apex, whereas the coronal threads achieved full osseointegration in bone (Fig. 6a).

The apex contacted the PDL, dentin, and the newly formed osteodentin that newly formed after implant placement (Fig. 6a–c); the PDL extended coronally until reaching the first areas of bone contact (Fig. 6a). Neoformed osteodentin was closely apposed to the rough surface of the apex (Fig. 6d); it appeared as a direct continuation of the osteodentin previously deposited during healing after the partial extraction (Fig. 6c).

### SEM-EDS observation

**Fig. 7:**
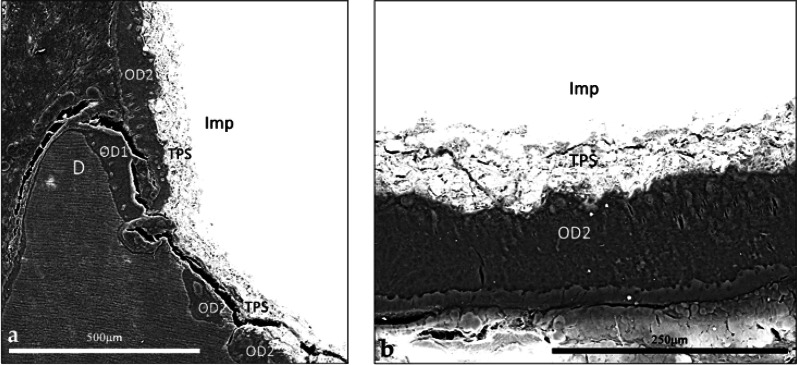
BSE-SEM micrographs at the implant-tissue interface. **a** View of the dentin-osteodentin interface and the close apposition of osteodentin to the implant surface, in direct continuation of the dentin. Note the neoformed tissue closely apposed against the rough TPS implant surface. The neoformed matrix displayed a deeper tone, indicative of lower mineralization than the dentin that appeared with its characteristic tubules (SEM, x250, scale bar 500 μm). **b** Close apposition of neoformed osteodentin against the TPS-coated apex. The implant appears plain white, the TPS coating displays its layered structure (SEM, x500, scale bar 250 μm). *D* dentin, *OD1* osteodentin formed following partial extraction, *OD2* osteodentin formed after implant placement, *TPS* titanium plasma-sprayed coating, *Imp* implant

**Fig. 8:**
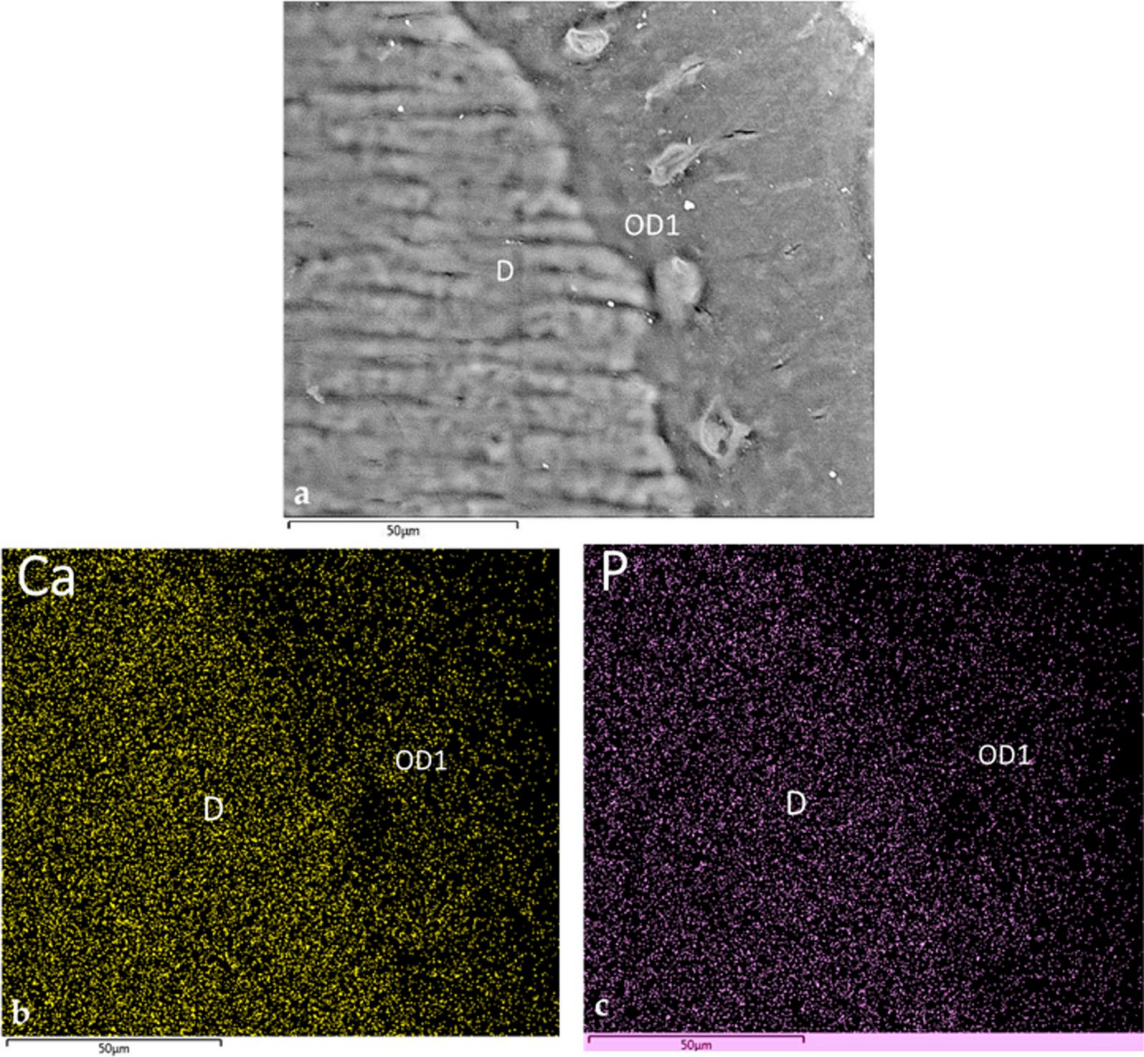
Elemental mapping of Ca and P of the dentin-osteodentin interface. **a** SEM micrograph in the backscattered mode at the interface between the dentin and the newly formed mineralized tissue. Note the orientation of the tubules of the dentin, the osteodentin and the marrow-like space (SEM, x1000, scale bar 50 μm). **b** Ca mapping of the interface between the dentin and the newly formed mineralized tissue. The neoformed tissue shows a reduced signal compared with the adjacent dentin. **c** P mapping of the same interface. The neoformed tissue shows a similarly reduced signal intensity. *D* dentin, *OD1* osteodentin formed after partial extraction

The micrographs highlighted the sharp transition between pristine dentin with its characteristic tubules and the newly formed osteodentin (Fig. 7a). The osteodentin was directly apposed to the implant apex without any intervening soft tissue (Fig. 7a and b). Ca and P mapping at the dentin–osteodentin interface confirmed a reduction in these mineral components within the newly formed matrix (Fig. 8a–c); this observation was consistent with the findings of the first implant.

### Implant #3 in contact with root debris, retrieved after 12 weeks

#### LM observation

**Fig. 9:**
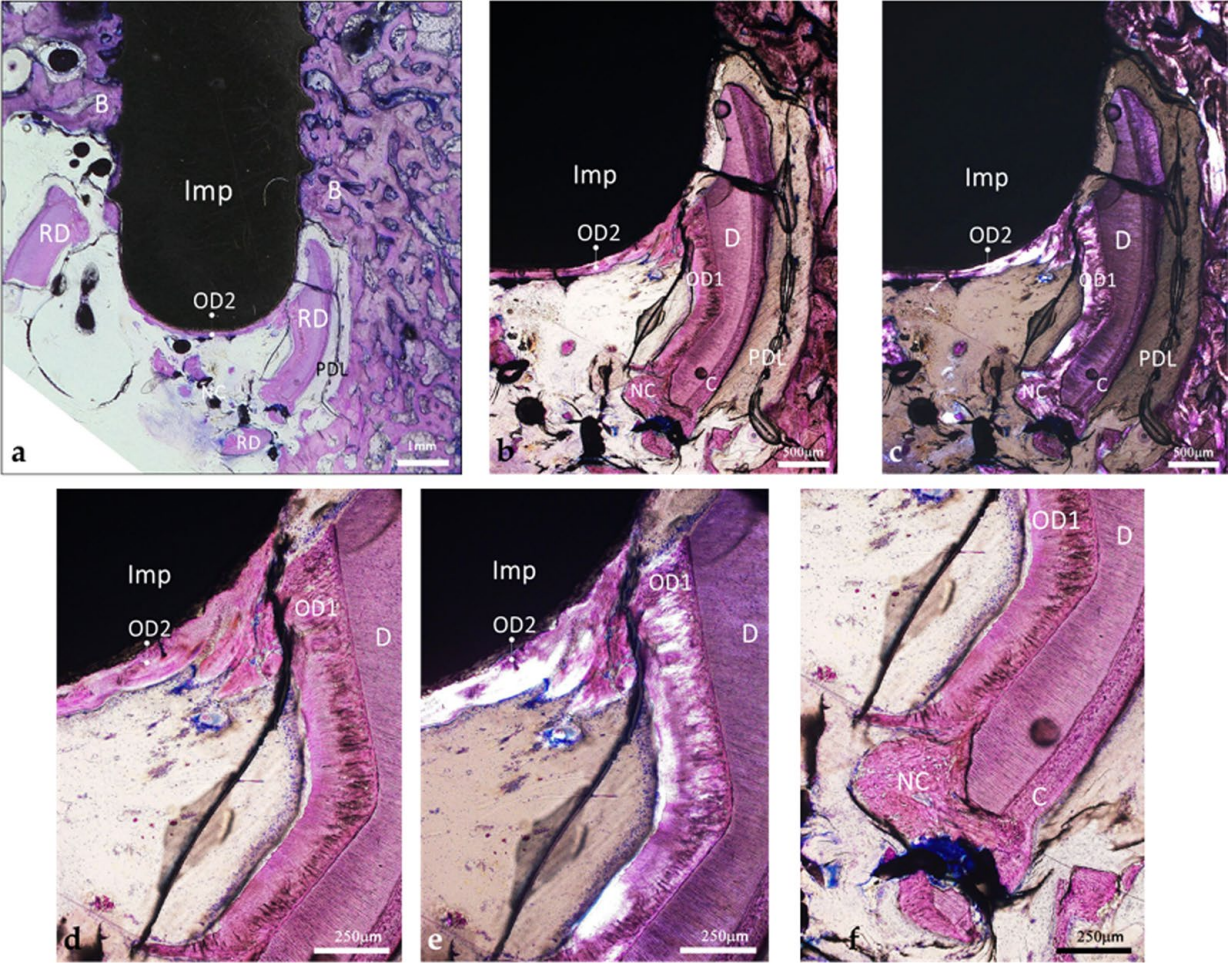
Histological micrograph of the implant in contact with debris of a retained root. **a** Only the implant apex is in contact with the debris of the retained root. Note the implant threads in contact with spongious bone, the various root debris with the PDL and apposition of a neoformed tissue at the apex (LM, objective x2, scale bar 1 mm). *B* bone, *RD* root debris, *PDL *periodontal ligament, *OD *osteodentin. **b** Close-up of the previous view showing the neoformed tissue in contact with the apex and its continuity with the reparative tertiary dentin (LM, objective x4, scale bar 500 μm). **c** Polarized-light observation of the same area. Each tissue displayed distinct birefringence. Note the distinct organizations of the collagen matrix of the mineralized tissues (PLM, objective x4, scale bar 500 μm). **d** Higher-magnification view of the apex showing the neoformed tissue in contact with the implant and its direct continuity with the reparative tertiary dentin (LM, objective x10, scale bar 250 μm). **e** Observation of the previous view under polarized light. Note the similar birefringence of the reparative tissue adjacent to the dentin and the tissue apposed to the apex (PLM, objective x10, scale bar 250 μm). **f** View of the lower part of the root debris. Note the dentin, the cementum, the PDL, the neocement that surrounds the dentin and the reparative dentin. The neocement remained confined to the root surface and did not extend toward or contact the reparative tissue apposed to the implant surface (LM, objective x10, scale bar 250 μm). *D* dentin, *C* cementum, *OD1* osteodentin formed after extraction, *OD2* osteodentin formed after implant placement, *NC *neocement, *PDL *periodontal ligament

In this case, the residual root fragments were contacted only by the implant apex (Fig. 9a); the implant threads were surrounded by bone and achieved osseointegration. A layer of osteodentin, newly deposited after implant placement, was evident along the apex surface; it was in continuity with the osteodentin formed after tooth extraction (Fig. 9b–e). Neocement was covering portions of exposed dentin; however, it remained spatially separated from the implant apex (Fig. 9f).

## Discussion

This report adds three distinct histological scenarios to the scarce literature dealing with implants that have been unintentionally engaging retained roots: (1) an implant fully embedded within the remains of a retained root, (2) an implant in partial contact to a retained root at the apex, and (3) an implant apex in contact with debris from a fractured root. To the best of our knowledge, complete encasing of a dental implant within a retained root has not been documented so far.

The most striking observation in this rare configuration was the direct and uninterrupted interface between the implant surface and the various mineralized tissues of the retained root, namely dentin, osteodentin and neocement. Osteodentin is the result of reparative dentinogenesis; it is a defense mechanism characterized by the rapid differentiation of odontoblast-like cells from progenitor/stem cells recruited at the injured site [26, 27]. Because this process is an emergency reaction, the resulting tissue does not form normal tubular dentin; instead, the newly differentiated cells produce a disorganized matrix resembling woven bone [26, 27] ultimately yielding atubular dentine [27]. This reparative matrix had developed over the retained root during the 3-month healing period following extraction; it was present at the time of implant placement and it was also newly deposited in response to implantation.

Alike bone, osteodentin displayed dynamic remodeling activity of the dentin along the implant threads. Noteworthy, when dentin was in direct contact with the implant surface, no visible cellular remodeling activity was detected at the 10-week timepoint; in contrast, areas containing small spaces, in the valleys between the threads or at the beveled apex, consistently reveled an active formation of mineral tissues. The present observation that mineralized tissue formation occurs preferentially in areas where space exists between the implant and surrounding tissues has been consistently reported for mineralized dental tissues [19–22]. A similar pattern of behavior has been documented at the bone-implant interface during early healing [28–30]. These findings align with the concept that creating microgaps between the implant surface and the surrounding bone [28–30] might stimulate biological integration.

Another remarkable finding concerns the behavior of the PDL. Although the retained roots preserved their PDL, no extension or proliferation over the entire implant surface was observed, an expansion that would have resulted in soft tissue encapsulation. Previous studies [16, 17] showed that PDL proliferation requires an open environment permissive for expansion; the close encasement of the implant by mineralized tissues likely acted as a physical barrier and prevented any extension of the rapidly forming PDL connective tissue [16–18].

The two other cases involved partial contact with retained root structures; they are consistent with previous observations that incidental apical contact with mineralized root remnants bordered by a PDL does not jeopardize implant osseointegration [19]. Apposition of newly formed osteodentin onto the implant apex was consistently observed without any interposed soft tissue, while the remainder of the implant surface demonstrated conventional osseointegration. Traditional osseointegration is defined by direct bone-to-implant contact; however, the present findings and earlier reports indicate that other mineralized tissues, such as dentin, neocement, and osteodentin, are also capable of forming intimate interfaces with implant surfaces [16–22]. Therefore, rather than characterizing this phenomenon as dentointegration [19], the more inclusive term *mineral integration* may be more appropriate [11]; this term encompasses all forms of mineralized tissue capable of interfacing directly with an implant.

With the development of the socket-shield technique (SST), intentional partial root retention has been explored histologically; these reports consistently describe apposition of neocement along the implant surface [20–22]. Only a single study by Schwarz et al. [19] documented osteodentin formation at the implant interface. The divergence between those findings and the present observations likely reflects differences in biological context: controlled root retention in SST versus traumatic injury and unintended root engagement in the present cases.

In the event of active injury such as root fracture, a reparative cascade is activated. This cascade involves the recruitment of pulp-derived or peri-radicular stem cells and initiates the release of pro-reparative signaling molecules such as transforming growth factor-beta (TGF-β) and bone morphogenetic proteins (BMPs). These signals trigger the differentiation of a new generation of odontoblast-like cells from progenitor/stem cells recruited to the site, leading to subsequent osteodentin deposition [31, 32]. In the account of Schwarz et al. [19], the implant was placed in contact with a vital root segment containing an active pulp and the authors attributed the observed tissue apposition to pulp-induced dentointegration of the titanium implant.

In the present report, the implants engaged fractured root fragments, with likely exposure either to pulpal remnants or reparative matrices produced earlier through the differentiation of odontoblast-like cells or dental pulp stem cells (DPSCs) into osteodentin-forming cells [26, 27]. Osteodentin is a tertiary reparative dentin; it is formed in response to injury, when pulpal or peri-radicular tissues activate mesenchymal cells to initiate repair [26, 27]. By contrast, the SST involves the intentional elective retention of a buccal root fragment while all the remains of the root, including the pulp, are meticulously removed [20–22, 33, 34]. Consequently, the deliberately retained buccal segment in SST behaves as a passive and biologically inert scaffold; it remains quiescent without triggering a reparative signaling cascade toward osteodentin formation. Instead, neocement forms along the implant surface in continuity with the residual cementum.

Noteworthy, this histological report deals with roots that were healthy and non-infected since they had been extracted for experimental purposes. In clinical practice, however, retained roots are typically associated with pathology, deep caries, fractures, periodontal and/or endodontic lesions. Therefore, if a similar biologic response were to be obtained in humans, it would likely occur only under infection-free conditions. Significantly, both the successful long-term cases reported by Szmukler-Moncler et al. [11] and the implant failures described by others [12–15] achieved biological stability and functional loading for several years, until pre-existing infection or chronic inflammation processes within the retained roots, possibly present but undetected, ultimately jeopardized implant function in the latter reports [12–15].

Several important limitations must be acknowledged in the present report: (1) the limited number of samples does not permit biologic or clinical extrapolation, (2) the 10- and 12-week time points capture early integration but do not allow assessment of long-term stability or functional adaptation, (3) pigs provide valuable preclinical models, however their root morphology and healing characteristics might differ from those of humans.

## Conclusion

This report contributes additional preclinical observations on tissue responses to implants unintentionally placed in contact with non-infected retained roots. Interactions between implant surfaces and mineralized dental tissues have been previously described [11, 35–39], however, documentation specifically addressing osteodentin at the implant interface remains limited to a single paper [19]. In the present specimens, complete encasement of an implant within mineralized dental tissues was observed, and histological analysis was consistent with the participation of osteodentin in the early modeling and remodeling events occurring at the interface during the initial healing phase.

Given the small number of cases and the experimental conditions under which they were obtained, no biologic or clinical conclusions can be drawn. Recent review articles [40–43] have highlighted the growing interest in this topic and they repeatedly emphasized the need for additional pre-clinical and clinical studies to clarify long-term clinical relevance.

## Data Availability

All data generated during this study are included in this article.
